# Identification of Histamine H_3_ Receptor Ligands Using a New Crystal Structure Fragment-based Method

**DOI:** 10.1038/s41598-017-05058-w

**Published:** 2017-07-06

**Authors:** Ida Osborn Frandsen, Michael W. Boesgaard, Kimberley Fidom, Alexander S. Hauser, Vignir Isberg, Hans Bräuner-Osborne, Petrine Wellendorph, David E. Gloriam

**Affiliations:** Department of Drug Design and Pharmacology, Faculty of Health and Medical Sciences, University of Copenhagen, Copenhagen, Denmark

## Abstract

Virtual screening offers an efficient alternative to high-throughput screening in the identification of pharmacological tools and lead compounds. Virtual screening is typically based on the matching of target structures or ligand pharmacophores to commercial or in-house compound catalogues. This study provides the first proof-of-concept for our recently reported method where pharmacophores are instead constructed based on the inference of residue-ligand fragments from crystal structures. We demonstrate its unique utility for G protein-coupled receptors, which represent the largest families of human membrane proteins and drug targets. We identified five neutral antagonists and one inverse agonist for the histamine H_3_ receptor with potencies of 0.7–8.5 μM in a recombinant receptor cell-based inositol phosphate accumulation assay and validated their activity using a radioligand competition binding assay. H_3_ receptor antagonism is of large therapeutic value and our ligands could serve as starting points for further lead optimisation. The six ligands exhibit four chemical scaffolds, whereof three have high novelty in comparison to the known H_3_ receptor ligands in the ChEMBL database. The complete pharmacophore fragment library is freely available through the GPCR database, GPCRdb, allowing the successful application herein to be repeated for most of the 285 class A GPCR targets. The method could also easily be adapted to other protein families.

## Introduction

G protein-coupled receptors (GPCRs) are membrane proteins activated by many diverse ligands including photons, ions, neurotransmitters, lipids, carbohydrates, nucleotides, amino acids, peptides (e.g. hormones) and proteins^1^. The GPCR family is with its ~800 genes among the largest families in the human genome^2^ and involved in most physiological processes^3, 4^. Their regulation of pathophysiology in diverse disease areas and accessibility at the cell surface have earned them a key role in medicine: more than 30% of the drug on the market target GPCRs^5^. 59 GPCRs have been drugged with small molecules^6^; the vast majority binding within a structurally conserved pocket within the transmembrane heptahelical bundle (7TM)^7^. Modelling of ligand-receptor complexes can now be performed with higher accuracy as a result of the increasing number of GPCR crystal structures, and has been evaluated in three community-wide ‘GPCR Dock’ assessments^8–10^.

Virtual screening is an efficient alternative to high-throughput screening to identify new ligands. This entails a preceding screening *in silico* of commercial or in-house compound catalogues against a target structure^11–13^ or ligand pharmacophore^14, 15^. We recently described the development of a new chemogenomics method to generate pharmacophores for GPCRs^16^. The method is based on the extraction of a reference library of crystal structure fragments, which are interacting moiety-residue pairs. The complete library of moiety-residue pairs has been made available through the GPCR database, GPCRdb^17, 18^. Although specific for GPCR ligand discovery, this is analogous to general resources that extract the residue-ligand fragment information from crystal structures^19–21^. Here, we describe the first application of this method to identify new histamine H_3_ receptor ligands.

The H_3_ receptor is found mainly in the CNS and is implicated in cognition, sleep regulation, feeding, memory, nociception and the sleep/wake cycle^22–24^. It functions both as a presynaptic autoreceptor, as well as a regulator of the release of other neurotransmitters; such as serotonin, dopamine and acetylcholine; which may be co-released with GABA in some neurons to control wakefulness^25^. H_3_ has a remarkably high level of constitutive activity^26^ also *in vivo* ^27^, and many of the classical H_3_ receptor antagonists have recently been found to be inverse agonists^22^. H_3_ is coupled to the G_i/o_ class of G proteins, leading to inhibition of the adenylyl cyclase and decreased cAMP generation^28, 29^. The H_3_ receptor has shown a potential therapeutic use in a number of CNS indications; ADHD, cognitive disorders, epilepsy, narcolepsy, neurodegeneration and pain^30, 31^; as well as in treating alcohol^32^ and eating^33^ behaviours (for general reviews see refs 22–24).

## Results

### Pharmacophore model and virtual screening hits

**Figure 1:**
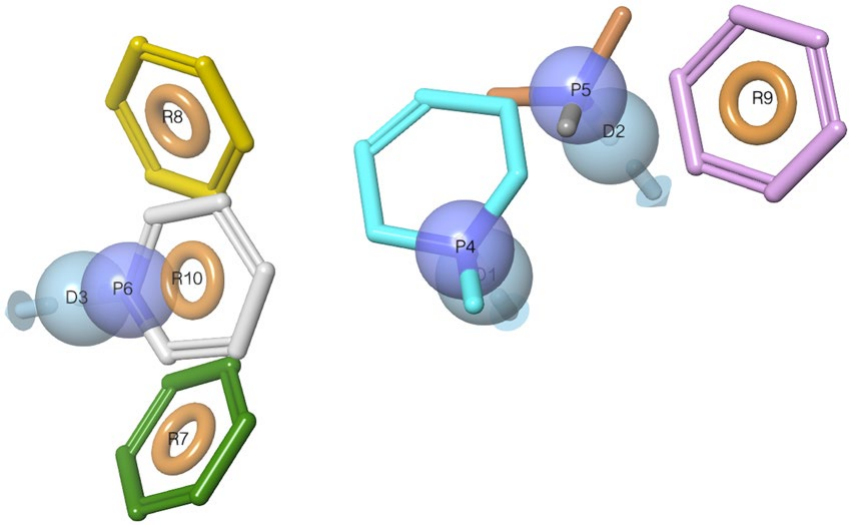
Histamine H_3_ receptor pharmacophore model used in the virtual screening for new ligands. The pharmacophore was based on a receptor residue – ligand moiety fragment library derived from GPCR crystal structures (Supplementary Table 1), and built with Phase^61^. The pharmacophore elements include three hydrogen bond donor (D1-3, light blue), three cation (P4-6, blue), three aromatic (R7, R9 and R10; orange) and one dual aromatic/hydrophobic (R8, orange) features.

The transmembrane domain of GPCRs can be aligned both in structure and sequence to identify the corresponding residue positions, which are below indexed using the GPCRdb scheme^34^. This is an evolution of the Ballesteros-Weinstein scheme^35^ modified to take into account helical bulges and constrictions observed in GPCR crystal structures. Matching of the H_3_ receptor sequence against our library of crystal structure fragments identified five conserved residues: W3.28, D3.32, Y3.33, Y6.51 and W6.48. A sixth residue, E5.46x461, could be added from reference ligands matched to the pharmacophore and docked into a H_3_ structure model. Apart from the residue, each fragment also has an interacting ligand moiety onto which the pharmacophore elements are placed. Figure 1 shows the final pharmacophore constructed from all the retrieved fragments, which are listed in Supplementary Table 1. The pharmacophore contains three hydrogen bond donor (D1-3), three cation (P4-6), three aromatic (R7, R9 and R10) and one dual aromatic/hydrophobic (R8 and R10) elements. The screening of this pharmacophore and subsequent hit clustering resulted in 44 compounds selected for purchase (Supplementary Chart 1).

The majority of the pharmacophore hits have long linkers between the two essential elements, the cationic P5 and aromatic R10, which is typical for antagonistic rather than agonistic aminergic ligands. Apart from the essential match to P5 (the site of the archetypical aminergic ligand cation interacting with the conserved D3.32), as many as 31 hits also matched P6, whereof only two matched the third element P4. The additional match to P6 is significant, as this feature, or rather its interacting residue E5.46x461, is not shared by the H_1–2_ receptors and therefore is likely to contribute to selectivity. Apart from the essential match to the aromatic element R10, about half (21) and a third (15) of the hits also matched R7 and R9, respectively (5 matched both). In contrast, R8 that is a dual hydrophobic/aromatic element matched only 3 aromatic moieties, but 23 hydrophobic. Finally, the number of matches for the hydrogen bonding elements reflected that of their adjacent cationic features, with 39, 33 and 5 matches for D3, D2 and D1, respectively.

### Histamine H_3_ receptor assay validation

**Figure 2:**
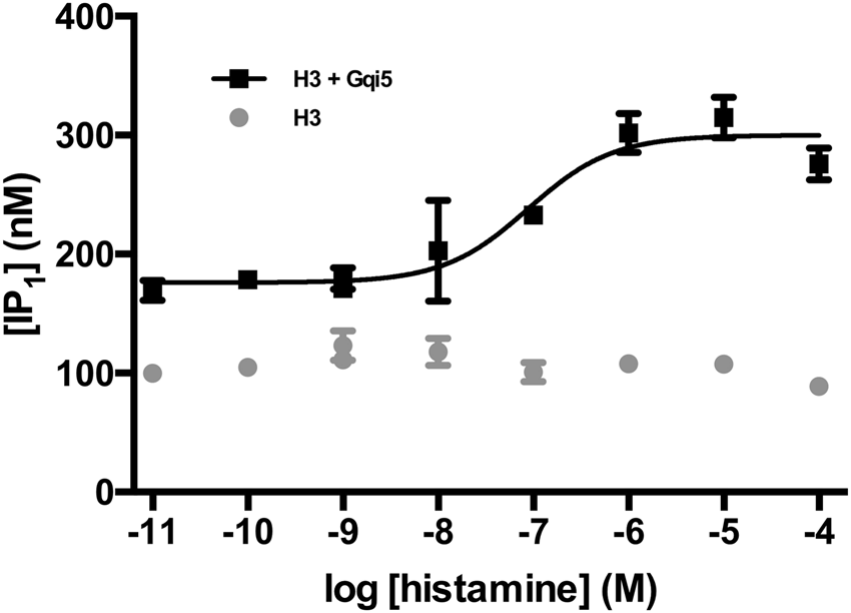
Assessment of histamine H_3_ receptor G_q_-coupled pharmacological assay using the IP_1_ accumulation assay measured by the HTRF IP-One assay. Concentration-dependent responses to histamine in H_3_ receptor expressing tsA201 cells transfected with (black) and without (grey) Gqi5. Data are means ± S.D. of a single representative experiment performed in triplicate. Two additional experiments gave similar results.

As shown in Fig. 2, the co-expression of H_3_ receptor and the chimeric G protein Gqi5 in tsA cells allowed for the generation of a histamine concentration-response curve in the IP-One assay with an EC_50_ value of 150 nM (pEC_50_ = 6.8 ± 0.4; n = 12). Furthermore, the reference antagonist thioperamide gave an IC_50_ value of 62 nM (pIC_50_ = 7.2 ± 0.3; n = 4), in the presence of EC_80_ of histamine (data not shown). Previous studies also using Gqi5 co-expression in HEK-derived cells reported values of 21.8 nM^36^ and 151 nM^37^ for histamine and thioperamide in mouse and human H_3_ receptors, respectively. The differences in EC_50_ and IC_50_; about 7-fold and 2-fold, respectively; may be attributed to differences in assay conditions (IP_1_ accumulation versus Ca^2+^ release), species used, and/or differences in expression system. To validate the suitability of the assay for high-throughput screening, the Z′-factors^38^ were determined, as described in methods. The Z′-factors determined were 0.5 and 0.3 for agonism and antagonism modes, respectively, which is indicative of an acceptable separation between the positive and the negative controls^38^. Thus, the assay was found to have adequate sensitivity, reproducibility and accuracy for the intended compound screening.

### Novel H_3_ receptor ligands

**Figure 3:**
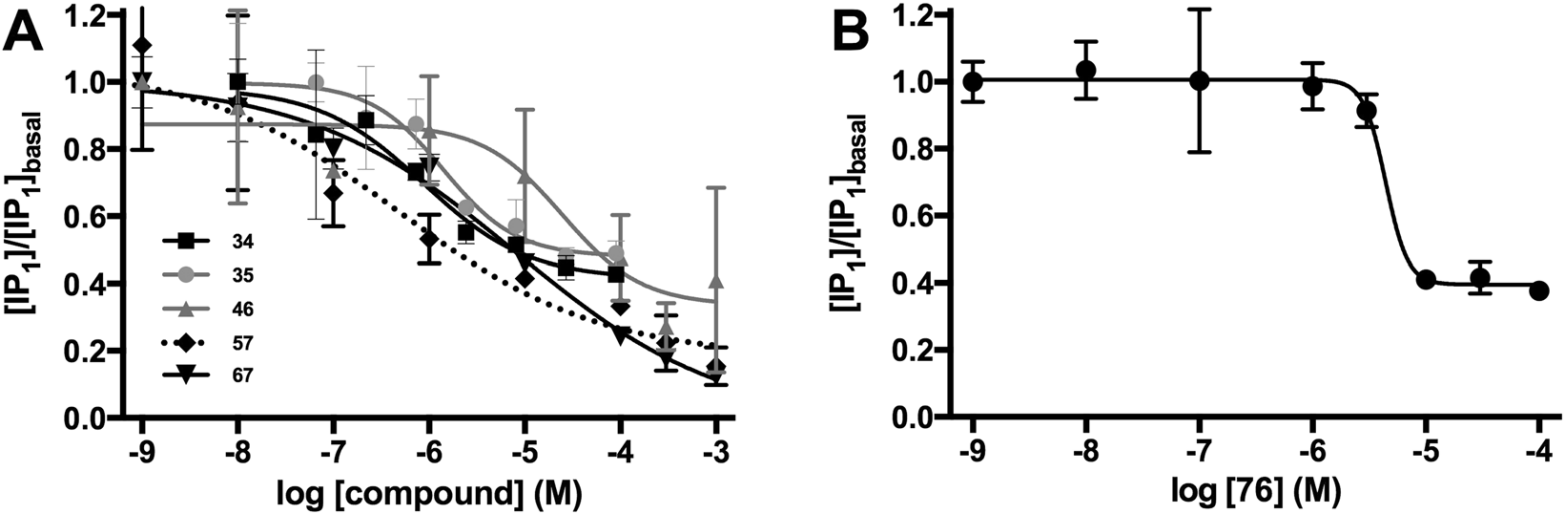
IP-One assay concentration-response curves. (**A**) Concentration-inhibition curves of the five identified neutral antagonists in the presence of histamine (EC_80_ concentration). **(B)** Inverse agonism of compound **76** (no histamine added). Data are normalized to the basal level of IP_1_ (fold response) in ligand buffer and are shown as means S.D. of a single representative experiment performed in triplicate. Due to solubility issues the lower plateau for the concentration-response curve of compounds **46**, **57** and **67** was constrained to the buffer value during curve fitting.

The first pharmacological assaying evaluated the selected 44 pharmacophore screening hits, and resulted in four antagonist hits; **21**, **34**, **35**, **42**. The second pharmacological assaying concerned 32 analogues of the four first hits (**21**: **45–48**, **34**: **49–54**, **35**: **55–72** and **42**: **73–76**), and resulted in four new antagonists **46**, **57**, **67** and **76**. Three seemingly active compounds from the first screen were excluded due to borderline activity (**20**) or an azaindolizine substructure (**9** and **44**), which can be fluorescent leading to a false positive response. Furthermore, after the secondary screen, compounds **21**, **42**, **64**, **68** and **75** were excluded because of the more stringent IC_50_ cut-off of 10 µM (Supplementary Table 2). The three antagonists **34**, **67** and **76** displayed inhibition below the basal level, and were therefore also tested as inverse agonists. Inverse agonistic activity was shown for **76** with an IC_50_ of 4.2 µM but not for **34** and **67**. In summary, we identified five antagonists (**34**, **35**, **46**, **57** and **67)** (Fig. 3A) and one inverse agonist (**76**) (Fig. 3B), with IC_50_ values ranging 0.66–8.5 µM (Table 1).

**Table 1:** Identified H_3_ receptor ligand structures, potencies and most similar ChEMBL ligand.

Cmpd	Structure	IC_50_ (µM)	pIC_50_ ± SEM	n	Ki (µM)	Affinity (pKi)	n	Most similar ChEMBL ligand	ECFP-4* similarity	Affinity (pKi)
34		0.66	6.2 ± 0.13	5	1.97	5.71 ± 0.17	5	ChEMBL 237514	0.26	6.1
35		3.3	5.5 ± 0.27	5	1.75	5.76 ± 0.05	4	ChEMBL 1923529	0.42	6.2
46		6.6	5.2 ± 0.37	3	6.07	5.22 ± 0.09	3	ChEMBL 2048591	0.24	7.3
57		5.2	5.3 ± 0.37	4	1.59	5.80 ± 0.14	3	ChEMBL 460313	0.37	6.9
67		8.5	5.1 ± 0.25	6	3.52	5.45 ± 0.19	3	ChEMBL 186456	0.36	5.5
76		2.7	5.6 ± 0.14	2	1.59	5.80 ± 0.05	3	ChEMBL 65849	0.26	8.0
	Inverse agonism activity	4.2	5.4 ± 0.068	3						

^*^ECFP-4 2D topological similarity.

**Figure 4:**
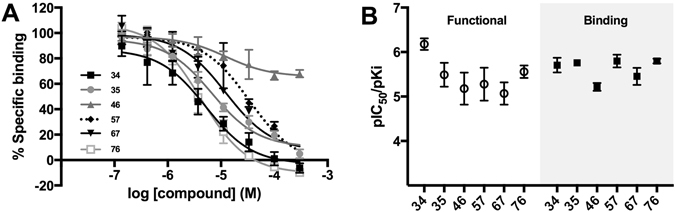
(**A**) Radioligand competition binding assay validates hits at the H_3_ receptor. Representative curves of the competitive binding of [^3^H]*N*-α-methylhistamine (0.3 nM) in the presence of various concentrations of compounds. Data points are shown as the mean ± S.D. of triplicate measurements. (**B**) The obtained affinities correlate with the potencies obtained in the functional assay. The potencies of the six most potent compounds obtained from the IP-One assay (*Functional*) shown as pIC_50_ compared with their affinities as determined in the radioligand competition binding assay (*Binding*). All data points are shown as the mean ± S.E.M, n = 2–5.

To confirm the results obtained from the functional studies, the six compounds (**34, 35, 46, 57, 67** and **76**) were also examined in a [^3^H]*N*-α-methylhistamine radioligand competition binding assay (Fig. 4A). Their *K*_i_ values ranged between 1.59–6.07 µM (Table 1) and were generally in good agreement with the potency observed in the functional study (Fig. 4B).

We assessed the novelty of the ligand structures by identifying the most similar H_3_ ligands in ChEMBL (Table 1). Ligand structures can be considered novel if their fingerprints have a Tanimoto Coefficient below 0.4^39^. **35** is just above this cut-off and **57** and **67** come close, whereas **34**, **46** and **76** are quite dissimilar to the known H_3_ ligands. The three least novel structures, share a fused triple ring core that in **35** contains a central imidazole, which is shared by histamine and many surrogate ligands. In contrast, the three novel ligands **34**, **46** and **76**, contain scaffolds that are distinct also among the herein described hit compounds. Apart from fused ring systems, all six ligands contain an amine that is positive ionisable, which was an essential match in the pharmacophore screening. Furthermore, **76** contains an amine β-hydroxyl and **46** a 4-aryl-piperidine that are known to be common functionalities in many aminergic and GPCR^40, 41^ ligands, respectively.

### Histamine receptor binding site selectivity hotspots

**Figure 5:**
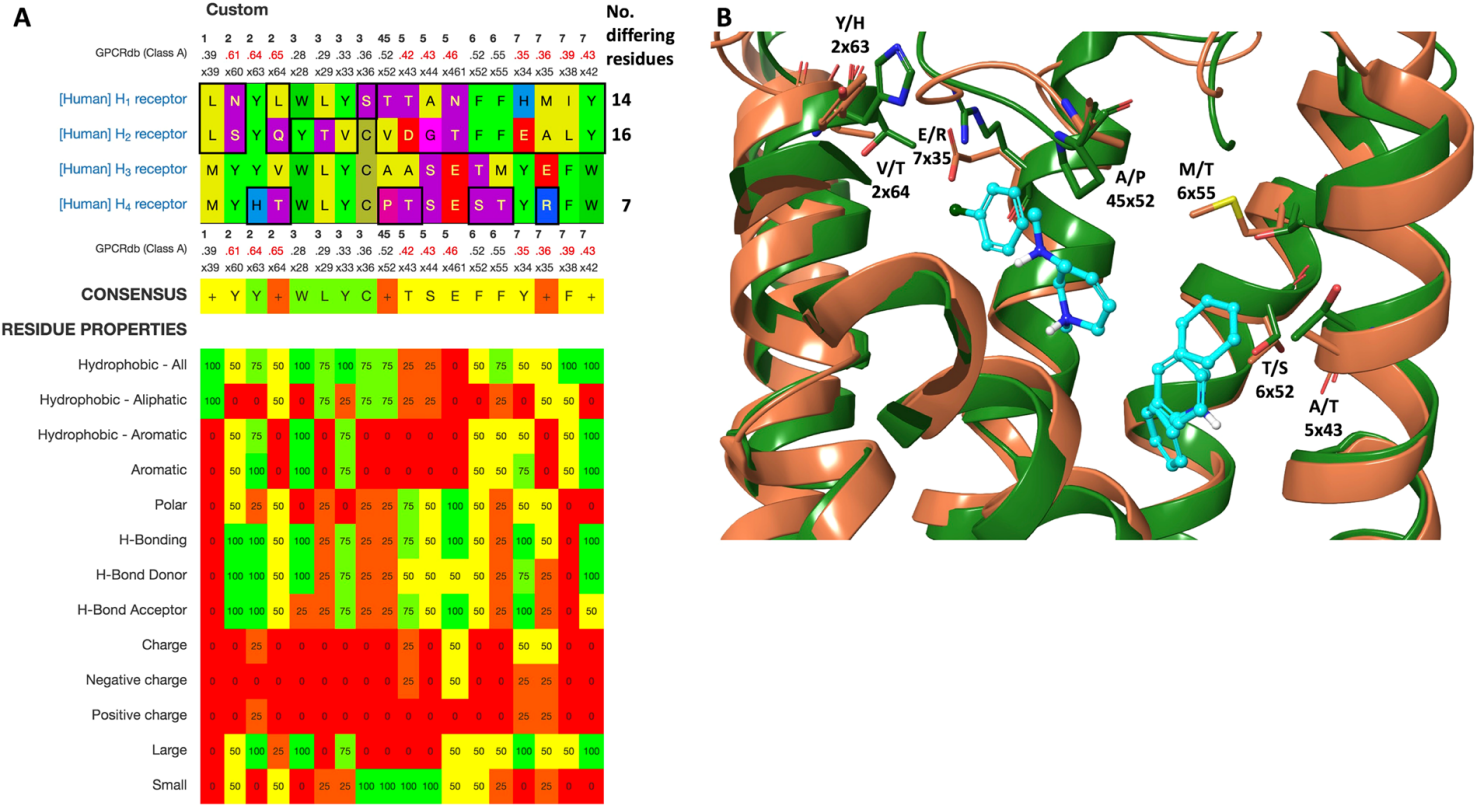
Histamine receptor subtype selectivity hotspots. (**A**) Sequence alignment of the non-conserved histamine receptor binding cavity residues that offer the most viable contact points for achieving subtype-selective ligands. Below the consensus sequence is shown also the residue properties, which when unique for the target may guide the choice of ligand substitution. (**B**) Superposition of the H3 target (brown) with the most homologous receptor, H4 (green). Pharmacophore fragments are displayed as balls and sticks (cyano) and selectivity hotspot residues as tubes.

Whereas experimental ligand selectivity was outside of the scope of this study, we compared the sequences and structures of the four histamine receptor subtypes to provide a rationale for future optimisation efforts. This showed that the H_1_, H_2_ and H_4_ anti-targets contain 14, 16 and 7 binding pocket residues, respectively, that differ from the target, H_3_ (Fig. 5A). As previously shown for the metabotropic glutamate receptors^42^, such residues represent the receptor selectivity hotspots that could be targeted by interactions with new ligand substitutions. Specifically, the analysis shows that selective analogues can be achieved by exploiting at least one H_3_-unique residue (V2x64, A45x52, A5x43, T6x52, M6x55 or E7x35) or a combination of residues that are conserved between some, but not all receptor subtypes (M1x39, Y2x60, W3x28, L3x29, Y3x33, C3x36, S5x44, E4x461, Y7x35, F7x38 or W7x42). Structural comparison of the H_3_ with the most homologous receptor, H_4_, shows that selectivity hotspots surround the pharmacophore fragments from all sides, except from TM3 in which all binding site residues are conserved (Fig. 5B). Hence, structure-based ligand optimisation of the H_3_ ligands obtained herein (and elsewhere) can access the receptor selectivity hotspots by a range of substitution vectors.

## Discussion

Our crystal structure fragment-based method^16^ led to the identification of five neutral antagonists and one inverse agonist for the histamine H_3_ receptor. Apart from their potential therapeutic value, neutral antagonists are interesting for pharmacological studies as they by definition inhibit both agonists and inverse agonists^43^. In contrast, no agonists were identified herein and this is expected for structure-based techniques that use a receptor template in the inactive state – we placed the fragments onto the transmembrane helix backbone of the H_1_ receptor crystal structure in complex with the inverse agonist doxepin (PDB: 3RZE)^44^. For agonist identification studies, the same library fragments applied herein should instead be superposed to an active state structure, such as the β_2_-adrenoceptor-Gs protein complex^45^. As the ligand binding site contracts upon activation^46^, such a pharmacophore could better accommodate agonists, which are typically smaller than antagonists. The six ligands identified herein display four distinct scaffolds, and in particular **34**, **46** and **76** were found to be novel when comparing to the known H_3_ receptor ligands in ChEMBL, which is the largest public database for bioactive drug-like small molecules. These could represent new chemical entries for drug discovery, and whereas we herein tested the commercially available analogues, it would be highly warranted to further explore these as starting points in the synthesis and optimization of a larger number of analogues.

This study represents the first proof-of-concept for the application of our crystal structure fragment-based method in a prospective virtual screening, and gave a hit rate of 8% (6/76 compounds with IC_50_ < 10 μM). The best ligand potency and affinity was 660 nM and 1.6 µM, respectively. In a recent study, Lepailleur *et al*. used a traditional ligand-based pharmacophore, not reporting a hit rate but leading to 41.6 nM affinity at the H_3_ receptor^14^. Sirci *et al*. combined ligand- and protein-based molecular fingerprinting methods, and achieved a very high hit rate, 62%, and a best affinity of 0.5 μM (which is also high affinity considering that these hits were all fragments)^47^. This suggests that there are several techniques that can lead to comparable results, and that rates are influenced by the extent to which diverse and novel scaffolds are selected. What sets this method apart is instead the advantage that ligands can be identified without the requirement for previously known ligands. If such data would have been incorporated (beyond the E5.46x461 fragment) or the structural novelty not enforced as a filter, it can be expected that the hit rate and affinity would have been higher. Another advantage observed in the application of the crystal structure-based method to the H_3_ receptor target is that its inference from other (crystallized) receptors yielded a larger number of pharmacophore elements – ten instead of four^14^ and five^47^, respectively. This offers unique opportunities to target medicinal chemistry substitutions in the ligand optimisation phase on the basis of crystallographic evidence.

The unique ability to infer pharmacophore elements suggests that the crystal structure-based pharmacophore method is likely to offer a bigger advantage when applied to other GPCR targets that lack known ligands or a closely homologous crystal structure. Klabunde *et al*. succeeded in the identification of C3a receptor ligands using a method based on fragments and pharmacophores^48^. A larger variety of studies have inferred whole scaffolds or ligands between targets, based on chemogenomic techniques that detect local similarities within the transmembrane binding pocket^49–56^. The ability to perform such binding pocket comparisons have greatly increased as an effect of the many high-quality templates in the form of GPCR crystal structures, many of which are in complex with ligands. In this analysis, the H_3_ receptor was used as a template to place residue backbone atoms onto transmembrane helices. This receptor displays a sequence similarity of 59% and 57% to the H_1_ receptor transmembrane bundle and the doxepin binding site (5 Å proximity), respectively. The fragments, defining the residue sidechains and ligand moieties, were instead inferred from a variety of aminergic receptors. For such SAR-borrowing it is crucial to use structure-based sequence alignments to ensure that the equivalent residues are correctly identified, but this is greatly complicated by a number of frequent structural distortions in GPCR helices, bulges and constrictions. Specifically, one residue, E5.46x461, in the applied fragments is located at the tip of a bulge in many aminergic GPCRs. We used the GPCRdb alignments, which take this into account by defining the corresponding residue structure/sequence positions by superposition of crystal structures, and appends a second correct generic residue number to the Ballesteros-Weinstein number^34^.

In the present study, we utilized co-transfection of the human histamine H_3_ receptor with the chimeric Gqi5 protein to channel receptor signalling into the Gq pathway. Others have successfully applied this strategy in the attempt of developing robust Gq-directed pharmacological assays for GPCRs in general^57^, as well as for the mouse^36^ and human^58^ H_3_ receptor. Instead of utilizing Gq coupling for measuring calcium release we chose to develop an HTRF-IP_1_ accumulation assay that offers high sensitivity in an efficient 384-well format^59^. This assay was successfully established and showed satisfactory Z′-factors taken into consideration that it was a transient expression of receptor and G protein. It is to our knowledge the first demonstration of applying the IP-One Tb Cisbio assay on the histamine H_3_ receptor. Hence, with these assays we were able to confirm histamine agonism and identify several novel antagonists and one inverse agonist, demonstrating a broad detection range and versatility.

The subsequent characterization of the most potent compounds in the radioligand competition binding assay provides evidence that the inhibitory activity seen in the IP-One assay was mediated via an interaction with the H_3_ receptor, as the potencies/affinities obtained in both assays generally correlate well (Fig. 4B). This was however not true for all the compounds, as compounds **21** and **68** were unable to compete with binding of [^3^H]*N-*α-methylhistamine in the binding assay despite having previously shown inhibitory activity in the IP-One assay. This conflicting data may be due to an interference with the IP-One assay or an unspecific event leading to an apparent inhibition, such as cytotoxicity. This underlines the value of validating hits after a screening with an assay based on another principle. Additionally, the binding assay allowed for a more precise determination of the compound potency rank order, as the variations in the pIC_50_ values from the IP-One assay was greater than the corresponding variation in the pKi values for the binding assay (Fig. 4B). Interestingly, some compounds (**42**, **44** and **46**) were unable to lower the binding of [^3^H]*N-*α-methylhistamine to non-specific binding levels, as would otherwise be expected for ligands competing for binding at the orthosteric site. It cannot be ruled out that the unprecedented number of (10) pharmacophore elements, whereof three cationic elements and four aromatic, allow for compounds to bind simultaneously with the radioligand, [^3^H]*N*-α-methylhistamine, which is relatively small. However, future studies would be needed to firmly establish if **42**, **44** and **46** bind in an allosteric binding site or there is another explanation (e.g. a secondary binding site for the radioligand).

### Conclusions and Future Perspectives

A variety of alternative approaches exist for virtual screening for new lead drugs and pharmacological tool compounds. This study provided the first proof-of-concept for our recent pharmacophore method based on crystal structure fragments. This method will benefit from more fragments as the number of GPCR crystal structures increase even further, also for the same receptor in complex with a diversity of ligands. We are currently working to implement this method on targets with less structural information, including orphan receptors, and the application in the wider community is facilitated by free matching of the fragments to any class A GPCR target in GPCRdb. Both H_3_ receptor inverse antagonists and (neutral) antagonists are of proven therapeutic value. The ligands identified herein are drug-like molecules with novel scaffolds that could serve as starting points for further lead optimization.

## Methods

### Construction of a H_3_ pharmacophore

A structure-based sequence alignment of the H_1_ and H_3_ receptors was downloaded from GPCRdb^17^, and a model of the H_3_ receptor transmembrane domain was built in Modeller^60^ using the H_1_ crystal structure (PDB: 3RZE)^44^ template. The pharmacophore was designed using our previously described crystal structure-based pharmacophore method^16^, which is based on the manual annotation of a library of structural fragments, pairs of a receptor residue and interacting ligand moiety, from GPCR crystal structure complexes. Herein, we uploaded our H_3_ receptor model to GPCRdb^17^ to identify conserved residues represented in this library, and to superpose the backbones of the corresponding fragments. The pharmacophore elements were placed using Phase^61^ at the highest density of the (multiple) fragment moieties. The vectors of the hydrogen bonding features were defined after optimization of ligand moiety – receptor residue interactions. Furthermore, the H_3_ pharmacophore was extended with an additional element not covered by the fragment library, but instead defined by matching reference ligands to the pharmacophore as well as docking them into a H_3_ structure model. The additional pharmacophore element represents a cationic ligand functionality that interacts with a Glu residue in position 5.46 × 461. This residue has been shown by mutagenesis studies be important for binding of imidazole- and pyridine-containing ligands, including histamine^62, 63^.

### Preparation of reference ligand and screening databases

Histamine H_3_ receptor reference ligands were downloaded from ChEMBL^64^ and the IUPHAR guide to pharmacology databases^4^. We used only the ligands with submicromolar dose-response affinity or activity values (*K*_i_, p*K*_i_, EC_50_, pEC_50_, IC_50_ and pIC_50_), and the highest assay confidence scores: 8 or 9. The screening database, eMolecules plus^65^, was prepared with LigPrep^66^ to desalt, add hydrogen atoms and generate tautomers, stereoisomers (max 32) and 3D conformations (max 10 ring conformations). Epik and the OPLS 2005 force field were applied to generate charge states at pH: 7.0 ± 1.0^66^. LigFilter was used to remove structures with reactive functional groups and match the properties (Supplementary Table 4) of the reference ligands^67^.

### Pharmacophore screening and hit selection

The Phase database, containing both reference ligands and screening compounds, was prepared with 100 maximum conformers, up to 10 conformations per rotatable bond, thorough conformational sampling, conformational variation of amide bonds and a maximum relative energy difference of 6.0 kcal/mol. A minimum of four matching pharmacophore elements was required and a preference was set for partial matches involving more sites. Hits were sorted by fitness score and clustered with Canvas^68^ to select diverse representative structures. As a secondary assessment of compound structures, we used SiteMap^69^ on a H_3_ structure model. After the first assaying round small structure-activity relationship analyses were conducted and the compounds sorted into lead ligand series. The selections of analogues were based on substructures drawn in MarvinSketch and queried n the eMolecules database loaded into Instant JChem (Marvin 5.12.3, 2014 and Instant JChem 6.2.0, 2014, ChemAxon, www.chemaxon.com).

### Histamine receptor binding site comparison

The H_1_ crystal structure and updated H_2–4_ homology models were downloaded from GPCRdb^17, 18^. An initial alignment of all class A GPCR ligand binding pocket residues was retrieved, also from GPCRdb. This alignment was first filtered to pinpoint the receptor subtype-differing positions, and subsequently by structural investigation to extract only those residues that may be accessible to ligand substitutions (Fig. 5A).

### Pharmacological assaying materials

Buffers and media for cell culturing were all purchased from Invitrogen (Paisley, United Kingdom) whereas non-enzymatic dissociation solution, HEPES (4-(2-hydroxyethyl)piperazine-1-ethanesulfonic acid) and additional assay buffer supplements were from Sigma-Aldrich (St. Louis, MO, USA). The IP-One Tb assay kit was purchased from Cisbio (Codolet, France). The human histamine H_3_ DNA (Genbank accession no. AF321910.1) in a pSI expression vector was identical to a previously used one^58^. The G protein Gqi5 was a kind gift from Dr. Evi Kostenis, University of Bonn, Germany. Histamine hydrochloride and thioperamide maleate were obtained from Sigma-Aldrich and Abcam Biochemicals (Cambridge, UK), respectively.

### Cell culture and transfections

Human tsA201 cells, a transformed HEK-293 cell line^70^, were cultivated in Dulbecco’s Modified Eagle’s Medium (DMEM) with GlutaMAX, supplemented with 10% foetal bovine serum, penicillin 100 U/ml and streptomycin 100 µg/ml. Subconfluent cells grown in a 100 mm dish were transfected with 5 μg DNA using the Polyfect transfection reagent using the protocol of the manufacturer (Qiagen, West Sussex, UK), however with half of the recommended PolyFect volumes. Co-transfections (ratio 4:1) were performed with either constructs expressing the human histamine H_3_ receptor together with an empty vector and chimeric G protein Gqi5, respectively.

### IP-One assay

We applied the highly sensitive high-throughput homogeneous time-resolved fluorescence (HTRF) technology^71^ for detection of IP-One generation using the Cisbio IP-One Tb assay kit, exactly as previously described^72^. In brief, on the day of assay, ligand solutions were prepared in ligand buffer (Hank’s Balanced Salt Solution (HBSS) containing 20 mM HEPES pH 7.4, 1 mM CaCl_2_, 1 mM MgCl_2_, 40 mM LiCl)) and added to the wells of a 384-well OptiPlate (PerkinElmer, Waltham, USA) in triplicates. When testing for antagonist activity the ligand buffer was supplemented with an EC_80_ concentration of histamine, and we used a final compound concentration of 20 µM and 2 µM for the first (virtual hits) and second (analogues) screening, respectively. Cell suspensions were added and incubated with ligands for 1 hour, followed by the addition of detection solution (IP-One Tb conjugate & Lysis Buffer +2.5% anti-IP_1_ cryptate Tb conjugate +2.5% IP_1_-d2 conjugate. 38:1:1) and incubation in the dark for one hour at room temperature. The plate was read on an EnVision 2104 Multilabel Reader (PerkinElmer) by exciting the wells with light of 340 nm and measuring the emission at 615 nm and 665 nm. The fluorescence resonance energy transfer ratios (665 nm/615 nm) were converted to IP_1_ concentrations by interpolating values from an IP_1_ standard curve generated from an IP_1_ calibration stock, provided by the manufacturer (Cisbio).

### [^3^H]*N-*α-methylhistamine radioligand competition binding assay

HEK-239T cells were grown to 70% confluency in 150 mm cell culture dishes and transfected with 8 µg human H_3_ receptor using the Polyfect transfection reagent as previously described^72^. After 48 hours, cells were washed from each cell culture dish with Dulbecco’s phosphate buffered saline and harvested using a cell scraper. The resulting cell suspension was then centrifuged and resuspended in 500 µL lysis buffer (50 mM Tris-HCl, pH 7.4). The cell lysate was centrifuged at 16,000 g for 20 minutes at 4 °C. The resultant membrane pellets were stored at −80 °C until use. Each pellet was resuspended in 500 µL binding buffer (50 mM Tris-HCl, 0.5 mM EDTA, pH 7.4), homogenized and centrifuged at 16,000 g for 15 minutes at 4 °C, and subsequently resuspended in 15 mL of binding buffer to a desired protein concentration of approximately 0.2 mg/mL as determined by the method of Bradford^73^. The membrane solution (30 µg protein per well) was incubated for 90 minutes in 250 µL of binding buffer, with or without compound, together with 0.3 nM [^3^H]*N-*α-methylhistamine at room temperature. Non-specific binding was defined as the radioligand bound to membrane solution incubated with 10 µM histamine. The binding reaction was terminated by rapid filtration through Whatman GF/C unifilters (PerkinElmer), and washed four times with ice-cold wash buffer (50 mM Tris-HCl, 10 mM MgCl_2_, 0.1 mM EDTA, pH 7.4) using a 96-well Packard FilterMate cell harvester (PerkinElmer). Finally, Microscint 0 scintillation liquid (PerkinElmer) was added to the dried filters, and the radioactivity quantified in a Packard TopCount NXT microplate Scintillation Counter (PerkinElmer).

### Data analysis

In the first (virtual) screening, hits were selected if they displayed inhibition values greater than 3 standard deviations above the inhibition mean of the set of tested compounds. In the second screening, we applied a more stringent hit criterion requiring an IC_50_ value lower than 10 µM. Data were analysed using GraphPad Prism 6 (GraphPad Software, San Diego, CA, USA). Concentration-response curves were fitted by nonlinear regression to equation (1):

1 $${\boldsymbol{R}}={{\boldsymbol{R}}}_{{\boldsymbol{\max }}}+\frac{{{\boldsymbol{R}}}_{{\boldsymbol{\max }}}-{{\boldsymbol{R}}}_{{\boldsymbol{\min }}}}{1+{10}^{(\mathrm{log}{\boldsymbol{I}}{{\boldsymbol{C}}}_{50}-{\boldsymbol{X}})\ast {{\boldsymbol{n}}}_{{\boldsymbol{H}}}}}$$

where X is the logarithm of the concentration, *R* is the response, *R*_max_ is the maximal response, *R*_min_ is the minimal response, IC_50_ is the concentration giving half-maximum reduction of the response, and n_H_ is the Hill coefficient, which describes the steepness of the curve. IP-One assay results are reported either as raw FRET in arbitrary units, as IP_1_ concentration (nM) or as fold over basal (normalized to the basal level of IP_1_ in ligand buffer [IP_1_]/[IP_1_]_basal_). The Z′-factors were calculated using equation (2):

2 $${\boldsymbol{Z}}{\boldsymbol{^{\prime} }}=1-\frac{3{{\boldsymbol{\sigma }}}_{{\boldsymbol{c}}+}+3{{\boldsymbol{\sigma }}}_{{\boldsymbol{c}}-}}{|{\mu }_{{\boldsymbol{c}}+}-{\mu }_{{\boldsymbol{c}}-}|}$$

Z′ = 1 − ((3σ_c+_ + 3σ_c−_)/|μ_c+_ − μ_c−_|), where σ_c+_ and σ_c−_ are the standard deviation of the positive control and the negative control, respectively, and μ_c+_ and μ_c−_ are the mean of the positive control and the negative control, respectively. Values were determined for both agonist mode (histamine alone) and antagonist mode (histamine + thioperamide) using buffer as negative control. The Z′ determination was performed on a separate plate (n = 10–14 for each condition) and not on the library screening plates themselves, which, however, all contained controls that confirmed that the assay worked.

The binding data was fitted by nonlinear regression to equation (3):

3 $${\boldsymbol{Y}}={{\boldsymbol{Y}}}_{{\boldsymbol{\max }}}+\frac{{{\boldsymbol{Y}}}_{{\boldsymbol{\max }}}-{{\boldsymbol{Y}}}_{{\boldsymbol{\min }}}}{1+{10}^{{\boldsymbol{X}}-{\boldsymbol{logI}}{{\boldsymbol{C}}}_{50}}}$$

where X is the logarithm of the concentration, *Y* is the specific binding, *Y*_max_ is the maximal specific binding, *Y*_min_ is the minimal specific binding, and IC_50_ is the concentration giving half-maximum reduction in specific binding. The obtained IC_50_ values were converted to *K*_i_ values by the Cheng-Prusoff equation, using a published *K*_d_ value, 0.15 nM for this receptor-radioligand pair^58, 74^.

## Electronic supplementary material

Supplementary information

## References

[1] Pierce KL, Premont RT, Lefkowitz RJ (2002). Seven-transmembrane receptors. Nature reviews. Molecular cell biology.

[2] Venter JC (2001). The sequence of the human genome. Science.

[3] Lagerstrom MC, Schioth HB (2008). Structural diversity of G protein-coupled receptors and significance for drug discovery. Nature reviews. Drug discovery.

[4] Pawson AJ (2014). The IUPHAR/BPS Guide to PHARMACOLOGY: an expert-driven knowledgebase of drug targets and their ligands. Nucleic Acids Res.

[5] Drews J (2000). Drug discovery: a historical perspective. Science.

[6] Congreve M, Langmead CJ, Mason JS, Marshall FH (2011). Progress in structure based drug design for G protein-coupled receptors. J. Med. Chem..

[7] Gloriam DE, Foord SM, Blaney FE, Garland SL (2009). Definition of the G protein-coupled receptor transmembrane bundle binding pocket and calculation of receptor similarities for drug design. J. Med. Chem..

[8] Kufareva I, Katritch V, Participants of GD, Stevens RC, Abagyan R (2014). Advances in GPCR modeling evaluated by the GPCR Dock 2013 assessment: meeting new challenges. Structure.

[9] Kufareva I (2011). Status of GPCR modeling and docking as reflected by community-wide GPCR Dock 2010 assessment. Structure.

[10] Michino M (2009). Community-wide assessment of GPCR structure modelling and ligand docking: GPCR Dock 2008. Nature reviews. Drug discovery.

[11] de Graaf C (2011). Crystal structure-based virtual screening for fragment-like ligands of the human histamine H(1) receptor. J. Med. Chem..

[12] Schmidt D, Bernat V, Brox R, Tschammer N, Kolb P (2015). Identifying modulators of CXC receptors 3 and 4 with tailored selectivity using multi-target docking. ACS Chem Biol.

[13] Rodriguez D, Brea J, Loza MI, Carlsson J (2014). Structure-based discovery of selective serotonin 5-HT(1B) receptor ligands. Structure.

[14] Lepailleur A (2014). Dual histamine H3R/serotonin 5-HT4R ligands with antiamnesic properties: pharmacophore-based virtual screening and polypharmacology. J Chem Inf Model.

[15] Sanders MP (2011). Snooker: a structure-based pharmacophore generation tool applied to class A GPCRs. J Chem Inf Model.

[16] Fidom K (2015). A new crystal structure fragment-based pharmacophore method for G protein-coupled receptors. Methods.

[17] Isberg V (2016). GPCRdb: an information system for G protein-coupled receptors. Nucleic Acids Res.

[18] Munk C (2016). GPCRdb: the G protein-coupled receptor database - an introduction. Br. J. Pharmacol..

[19] Wass MN, Kelley LA, Sternberg MJ (2010). 3DLigandSite: predicting ligand-binding sites using similar structures. Nucleic Acids Res.

[20] Tang GW, Altman RB (2014). Knowledge-based Fragment Binding Prediction. PLoS. Comput. Biol..

[21] Wang L, Xie Z, Wipf P, Xie X-Q (2011). Residue Preference Mapping of Ligand Fragments in PDB. J. Chem Inf. Model..

[22] Leurs R, Bakker RA, Timmerman H, de Esch IJ (2005). The histamine H3 receptor: from gene cloning to H3 receptor drugs. Nature reviews. Drug discovery.

[23] Schwartz JC (2011). The histamine H3 receptor: from discovery to clinical trials with pitolisant. Br. J. Pharmacol..

[24] Berlin M, Boyce CW, Ruiz Mde L (2011). Histamine H3 receptor as a drug discovery target. J. Med. Chem..

[25] Yu X (2015). Wakefulness Is Governed by GABA and Histamine Cotransmission. Neuron.

[26] Wieland K (2001). Constitutive activity of histamine h(3) receptors stably expressed in SK-N-MC cells: display of agonism and inverse agonism by H(3) antagonists. J. Pharmacol. Exp. Ther..

[27] Morisset S (2000). High constitutive activity of native H3 receptors regulates histamine neurons in brain. Nature.

[28] Clark EA, Hill SJ (1996). Sensitivity of histamine H3 receptor agonist-stimulated [35S]GTP gamma[S] binding to pertussis toxin. Eur. J. Pharmacol..

[29] Clark MA, Korte A, Egan RW (1993). Guanine nucleotides and pertussis toxin reduce the affinity of histamine H3 receptors on AtT-20 cells. Agents Actions.

[30] Gemkow MJ (2009). The histamine H3 receptor as a therapeutic drug target for CNS disorders. Drug Discov Today.

[31] Bhowmik M, Khanam R, Vohora D (2012). Histamine H3 receptor antagonists in relation to epilepsy and neurodegeneration: a systemic consideration of recent progress and perspectives. Br. J. Pharmacol..

[32] Panula P, Nuutinen S (2011). Histamine and H3 receptor in alcohol-related behaviors. J. Pharmacol. Exp. Ther..

[33] Passani MB, Blandina P, Torrealba F (2011). The histamine H3 receptor and eating behavior. J. Pharmacol. Exp. Ther..

[34] Isberg V (2015). Generic GPCR residue numbers - aligning topology maps while minding the gaps. Trends Pharmacol. Sci..

[35] Ballesteros JA, Weinstein H (1995). [19] Integrated methods for the construction of three-dimensional models and computational probing of structure-function relations in G protein-coupled receptors. Methods Neurosci.

[36] Chen J, Liu C, Lovenberg TW (2003). Molecular and pharmacological characterization of the mouse histamine H3 receptor. Eur. J. Pharmacol..

[37] Esbenshade TA (2004). Pharmacological and behavioral properties of A-349821, a selective and potent human histamine H3 receptor antagonist. Biochem. Pharmacol..

[38] Zhang JH, Chung TD, Oldenburg KR (1999). A Simple Statistical Parameter for Use in Evaluation and Validation of High Throughput Screening Assays. Journal of biomolecular screening.

[39] Wawer M, Bajorath J (2010). Similarity-potency trees: a method to search for SAR information in compound data sets and derive SAR rules. J Chem Inf Model.

[40] Duarte CD, Barreiro EJ, Fraga CA (2007). Privileged structures: a useful concept for the rational design of new lead drug candidates. Mini Rev Med Chem.

[41] Patchett, A. A. & Nargund, R. P. In *Annu. Rep. Med. Chem*. Vol. 35 (eds William K. Hagmann & Annette M. Doherty) Ch. 26, 289–298 (Academic Press, 2000).

[42] Harpsoe K (2015). Selective Negative Allosteric Modulation Of Metabotropic Glutamate Receptors - A Structural Perspective of Ligands and Mutants. Scientific reports.

[43] Milligan G (2003). Constitutive activity and inverse agonists of G protein-coupled receptors: a current perspective. Mol. Pharmacol..

[44] Shimamura T (2011). Structure of the human histamine H1 receptor complex with doxepin. Nature.

[45] Rasmussen SG (2011). Crystal structure of the beta2 adrenergic receptor-Gs protein complex. Nature.

[46] Tehan BG, Bortolato A, Blaney FE, Weir MP, Mason JS (2014). Unifying family A GPCR theories of activation. Pharmacol. Ther..

[47] Sirci F (2012). Virtual fragment screening: discovery of histamine H3 receptor ligands using ligand-based and protein-based molecular fingerprints. J Chem Inf Model.

[48] Klabunde T, Giegerich C, Evers A (2009). Sequence-derived three-dimensional pharmacophore models for G-protein-coupled receptors and their application in virtual screening. J. Med. Chem..

[49] Garland, S. L. & Gloriam, D. E. A ligand’s view of target similarity: chemogenomic binding site-directed techniques for drug discovery. *Curr Top Med Chem* **11**, 1872–1881, doi:BSP/CTMC/E-Pub/-000110-11-13 [pii] (2011). 10.2174/156802611796391276 21470171

[50] Garland, S. L. & Gloriam, D. E. Methods for the successful application of chemogenomics to GPCR drug design. *Curr Top Med Chem* **11**, 1870–1871, doi:BSP/CTMC/E-Pub/-000105-11-13 [pii] (2011). 10.2174/156802611796391294 21470176

[51] Gloriam DE (2013). Chemogenomics of allosteric binding sites in GPCRs. Drug discovery today. Technologies.

[52] Jacoby E (2011). Computational chemogenomics. Wiley Interdisciplinary Reviews: Computational Molecular Science.

[53] Martin RE, Green LG, Guba W, Kratochwil N, Christ A (2007). Discovery of the first nonpeptidic, small-molecule, highly selective somatostatin receptor subtype 5 antagonists: a chemogenomics approach. J. Med. Chem..

[54] Frimurer, T. M. & Hogberg, T. Drug design of GPCR ligands using physicogenetics and chemogenomics–principles and case studies. *Curr Top Med Chem* **11**, 1882–1901, doi:BSP/CTMC/E-Pub/-000107-11-13 [pii] (2011). 10.2174/156802611796391258 21470174

[55] Rognan D (2007). Chemogenomic approaches to rational drug design. Br. J. Pharmacol..

[56] Kratochwil NA (2011). G protein-coupled receptor transmembrane binding pockets and their applications in GPCR research and drug discovery: a survey. Curr Top Med Chem.

[57] Kostenis E (2002). Potentiation of GPCR-signaling via membrane targeting of G protein alpha subunits. J. Recept. Signal Transduct. Res..

[58] Wellendorph P (2002). Molecular cloning and pharmacology of functionally distinct isoforms of the human histamine H(3) receptor. Neuropharmacology.

[59] Norskov-Lauritsen L, Thomsen AR, Brauner-Osborne H (2014). G protein-coupled receptor signaling analysis using homogenous time-resolved Forster resonance energy transfer (HTRF(R)) technology. International journal of molecular sciences.

[60] Eswar, N. *et al*. Comparative protein structure modeling using MODELLER. *Curr prot protein sci* Chapter 2, Unit 2.9, doi:10.1002/0471140864.ps0209s50 (2007). 10.1002/0471140864.ps0209s50 18429317

[61] Dixon SL (2006). PHASE: a new engine for pharmacophore perception, 3D QSAR model development, and 3D database screening: 1. Methodology and preliminary results. J. Comput. Aided Mol. Des..

[62] Shin N (2002). Molecular modeling and site-specific mutagenesis of the histamine-binding site of the histamine H4 receptor. Mol. Pharmacol..

[63] Uveges AJ (2002). The role of transmembrane helix 5 in agonist binding to the human H3 receptor. J. Pharmacol. Exp. Ther..

[64] Gaulton A (2012). ChEMBL: a large-scale bioactivity database for drug discovery. Nucleic Acids Res.

[65] eMolecules Plus Database, http://emolecules.com/ (2014).

[66] Greenwood JR, Calkins D, Sullivan AP, Shelley JC (2010). Towards the comprehensive, rapid, and accurate prediction of the favorable tautomeric states of drug-like molecules in aqueous solution. J. Comput. Aided Mol. Des..

[67] Small-Molecule Drug Discovery Suite v. 2014-2 (Schrödinger, LLC, New York, NY, 2014).

[68] Duan J, Dixon SL, Lowrie JF, Sherman W (2010). Analysis and comparison of 2D fingerprints: insights into database screening performance using eight fingerprint methods. J. Mol. Graph. Model..

[69] Halgren TA (2009). Identifying and characterizing binding sites and assessing druggability. J Chem Inf Model.

[70] DuBridge RB (1987). Analysis of mutation in human cells by using an Epstein-Barr virus shuttle system. Mol. Cell. Biol..

[71] Degorce F (2009). HTRF: A technology tailored for drug discovery - a review of theoretical aspects and recent applications. Current chemical genomics.

[72] Jacobsen SE (2013). Delineation of the GPRC6A receptor signaling pathways using a mammalian cell line stably expressing the receptor. J. Pharmacol. Exp. Ther..

[73] Bradford MM (1976). A rapid and sensitive method for the quantitation of microgram quantities of protein utilizing the principle of protein-dye binding. Anal. Biochem..

[74] Lovenberg TW (1999). Cloning and Functional Expression of the Human Histamine H3 Receptor. Mol. Pharmacol..

