# Supplementary material for: Identification of Histamine H3 Receptor Ligands Using a New Crystal Structure Fragment-based Method
Source: Sci Rep. 2017 Jul 6;7:4829. doi: 10.1038/s41598-017-05058-w (PMC5500575; doi:10.1038/s41598-017-05058-w)
Supplement: Supplementary file 1 — Supplementary information [file 41598_2017_5058_MOESM1_ESM.pdf]

Supplementary Information

Identification of Histamine H_3 Receptor Ligands Using a New Crystal Structure Fragment-based Method

Ida Osborn Frandsen[#], Michael W. Boesgaard[#], Kimberley Fidom, Alexander S. Hauser, Vignir Isberg, Hans Bräuner-Osborne, Petrine Wellendorph[*] and David E. Gloriam[*]

Department of Drug Design and Pharmacology, Faculty of Health and Medical Sciences, University of Copenhagen, Denmark

[#]These authors contributed equally
* Corresponding authors.

Supplementary Table 1: The fragments used to build the H_3 pharmacophore. The selected representative fragments for H_3 yielded 9 pharmacophore elements and a tenth, for 5.46x461, was added based on docking of reference ligand structures in a H_3 structure model. The aromatic pharmacophore elements are based on multiple fragments and were manually placed in their midst. The pharmacophore element types are abbreviated; Ar: aromatic, HBA: hydrogen bond acceptor, HBD: hydrogen bond donor, HYD: hydrophobic and ICT: ionic cation. *Residue positions are indexed using the GPCRdb scheme for generic residue numbering[1], which is the Ballesteros-Weinstein scheme[2] adjusted for helical bulges and constrictions observed in crystal structures.

Residue Position*	GPCR	PDB ID	Ligand Moiety	Element (Fig. 1)	Element Type	Size (Å) / Tolerance
**W3.28**	ADRB1	2Y00, 4AMJ	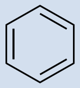	R9	Ar	1.5
**D3.32**	5-HT1B	4IAR	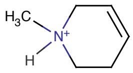	D1	HBD	1.0
				P4	ICT	0.75
	ADRB1	2Y03	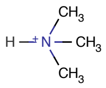	D2	HBD	1.0
				P5	ICT	0.75
**Y3.33**	M3	4DAJ	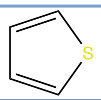	R8	Ar / HYD	1.5
	M2	3UON	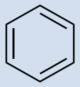			
	H1	3RZE				
**Y6.51**	ADRB1	2Y00, 2Y03, 2T04				
**E5.46x461**	H3	-	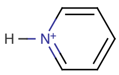	P6	ICT	0.75
				D3	HBD	1.0
				R10	Ar	1.5
**W6.48**	H1	3RZE	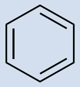	R7	Ar	1.5

Supplementary Table 2: Additional results from the IP-one assay

Compound	pIC_{50}±SEM	IC_{50} (μM)	n
**21**	4.68±0.10	20.9	5
**42**	4.38±0.11	42.1	5
**64**	3.58±0.17	261	4
**68**	4.66±0.20	22.0	2
**75**	3.92±0.18	121	3

Supplementary Table 3: Additional results from the radioligand competition binding assay

Compound	pKi±SEM	Ki (μM)	n
**9**[c]	5.76±0.25	1.75	3
**20**[c]	5.37±0.12	4.24	3
**21**[a]	-	>50.0	3
**42**[b]	4.83±0.08	14.8	4
**44**[b]	5.55±0.06	2.82	3
**64**	4.06±0.11	86.7	3
**67**	5.45±0.19	3.52	3
**68**[a]	-	>300	3
**75**	5.32±0.04	4.82	3

a Compound had little to no effect on radiolabeled ligand binding in the concentrations examined.
b Compound unable to outcompete radiolabeled ligand to NSB levels (non-competitive interaction).
c A continued decrease in potency observed upon repeated experimentation, possibly due to compound breakdown.
Ki values obtained by the method of Cheng-Prusoff using a Kd value of 0.15 nM for [3H]N-α-methylhistamine.

Supplementary Table 4: Filtering criteria for the eMolecule compounds

Parameter	Range
Molecular weight	200 - 500
Rotatable bonds	2 - 10
Rings	≤ 5
Aromatic carbons	≥ 6
Aliphatic rings	≤ 2
State penalty (Epik)	≤ 2
Reactive groups (#rtvFG)	0
Polar surface area (PSA)	< 150
Hydrophilic component of the solvent accessible surface area (FISA)	7 - 330
Predicted solubility (PlogS)	-6 - 0.5
Predicted logP (PlogPo/w)	< 5
Hydrogen bond acceptors	1 - 10
Hydrogen bond donors	1 - 5
Charged donor groups	≥ 1
Charged amines	1 - 3
Neutral amines	≤ 1
Total charge	2
Number of negative atoms	0

Supplementary Chart 1: Structures of the purchased pharmacophore hits (1-44) and primary assaying hit analogs (45-76). The 2D structures were drawn in ChemBioDraw v. 14.0.

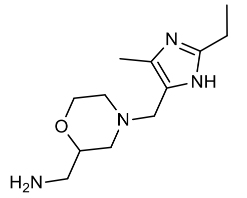
1
ID: 29497777

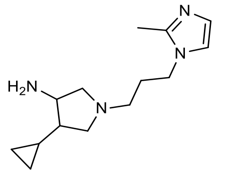
2
ID: 86897757

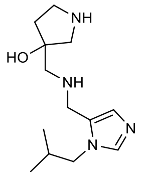
3
ID: 68385071

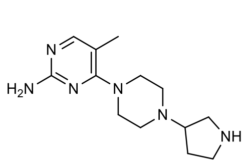
4
ID: 23747015

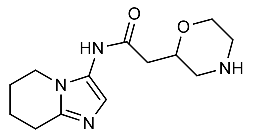
5
ID: 23988161

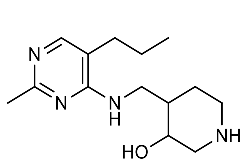
6
ID: 67683407

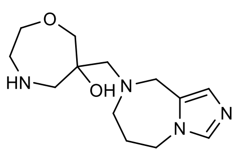
7
ID: 61398030

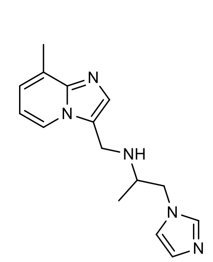
8
ID: 66670800

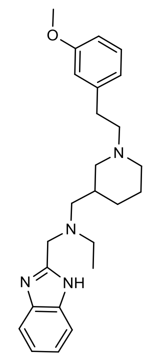
9
ID: 25762662

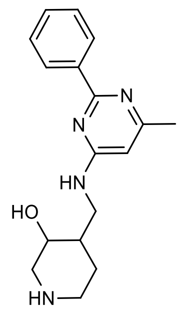
10
ID: 59853882

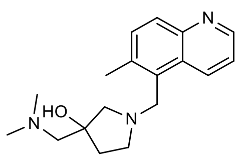
11
ID: 30502945

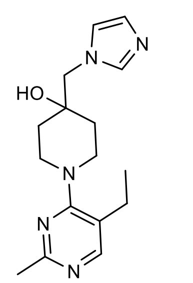
12
ID: 81492679

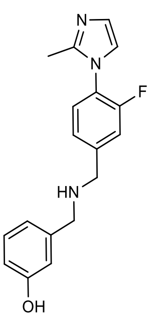
13
ID: Z973084186

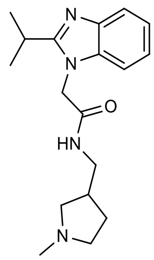
14
ID: 64477273

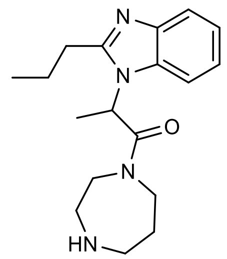
15
ID: 84684520

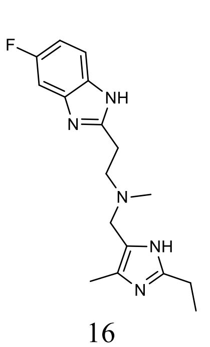
16
ID: 42630598

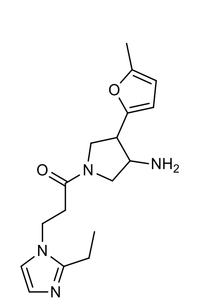
17
ID: 42691631

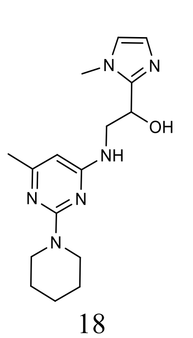
18
ID: 73832942

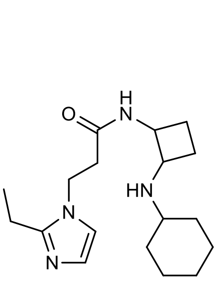
19
ID: 13698650

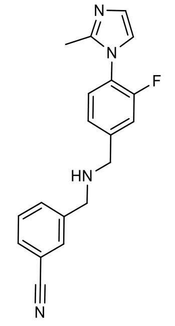
20
ID: Z840088398

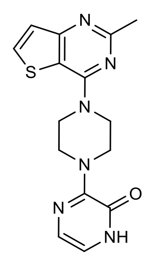

21
ID: 83352918

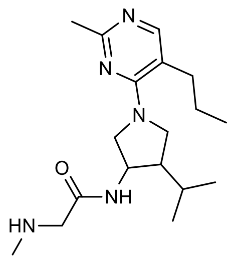

22
ID: 17412505

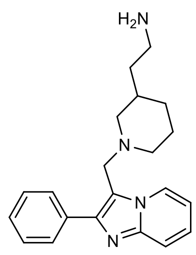

23
ID: 96256639

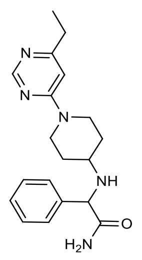

24
ID: Z1333147743

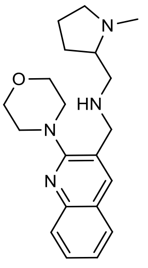

25
ID: Z840220692

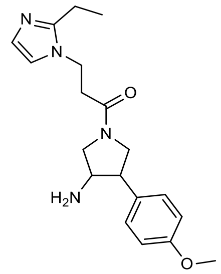

26
ID: 51422595

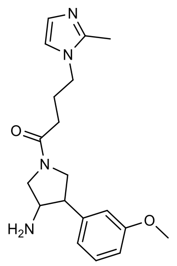

27
ID: 80792003

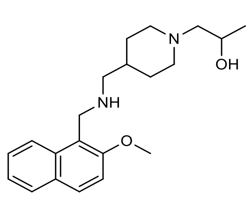

28
ID: Z1139559799

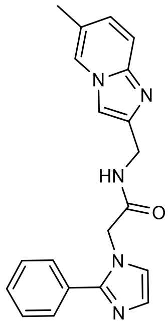

29
ID: 52135995

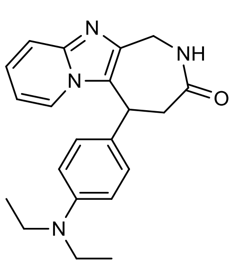

30
ID: 23270094

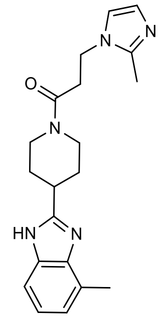

31
ID: 20073354

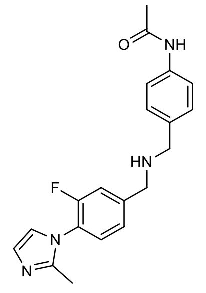

32
ID: Z840089412

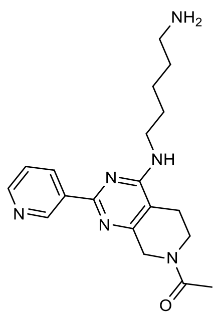

33
ID: 74699548

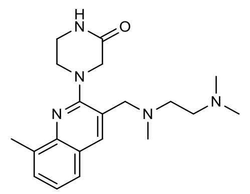

34
ID: 82687728

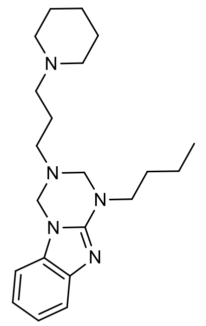

35
ID: Z107728962

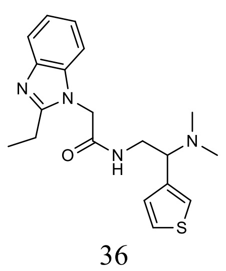

36
ID: Z343568958

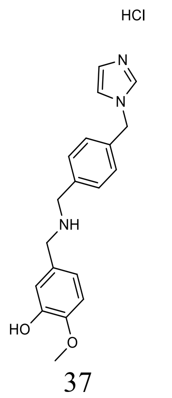

37
ID: Z401947192

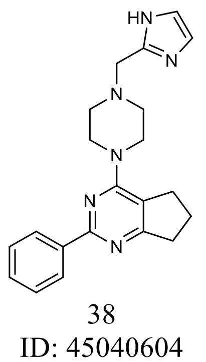

38
ID: 45040604

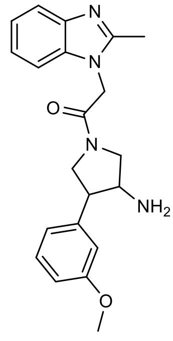

39
ID: 43911446

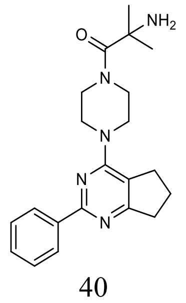

40
ID: 67481612

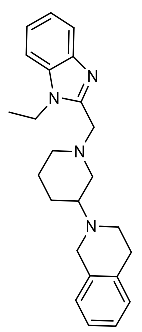
41
ID: 73088155

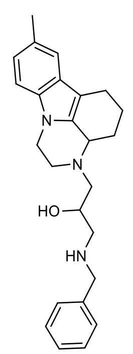
42
ID: STK530530

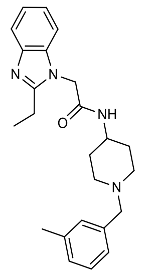
43
ID: PB653173258

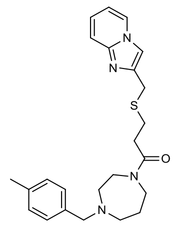
44
ID: Z371469800

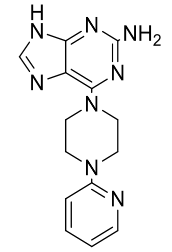
45
ID: STOCK6S-35722

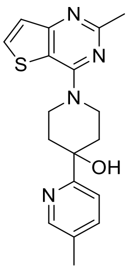
46
ID: 14144947

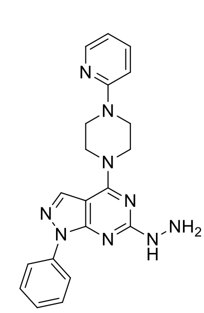
47
ID: STOCK3S-02044

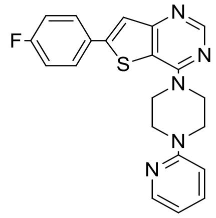
48
ID: Z31252931

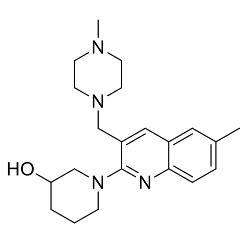
49
ID: 99314199

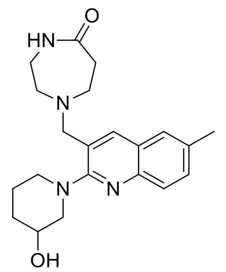
50
ID: 87627023

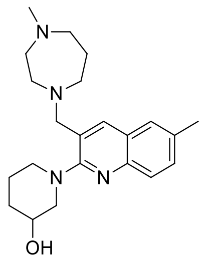
51
ID: 42681880

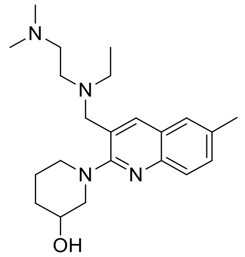
52
ID: 90452752

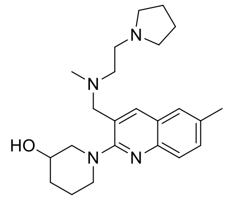
53
ID: 38644162

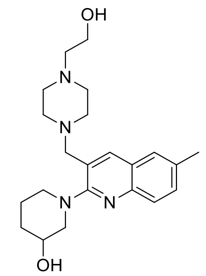
54
ID: 84865704

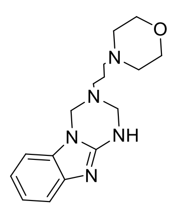
55
ID: STK167540

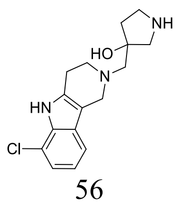
56
ID: 48840451

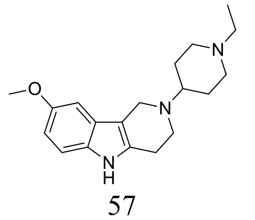
57
ID: 90940961

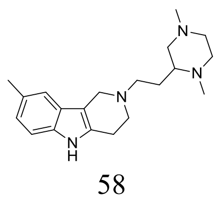
58
ID: 49988324

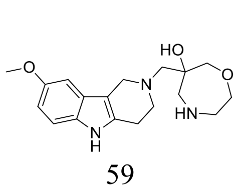
59
ID: 11893028

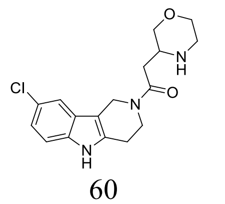
60
ID: 65933140

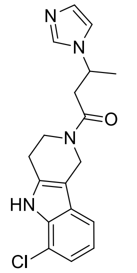
61

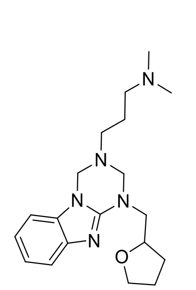
62

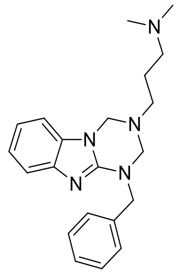
63

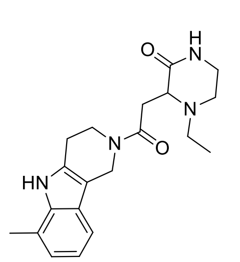
64

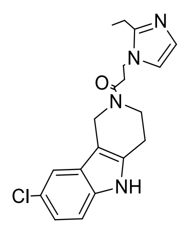
65

ID: 68828179 ID: STK790098 ID: STK356705 ID: 21263926 ID: 13575804

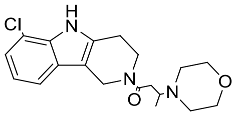

66

ID: 49065533

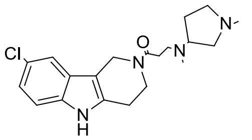

67

ID: 97882939

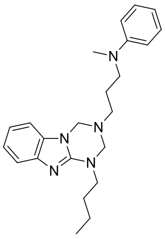

68

ID: Z104342908

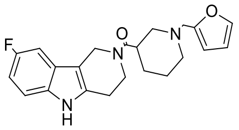

69

ID: 90400697

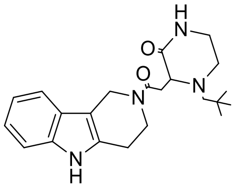

70

ID: 27779432

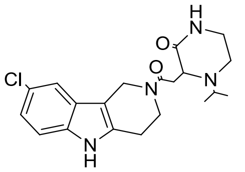

71

ID: 61725337

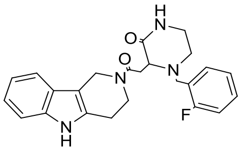

72

ID: 13841841

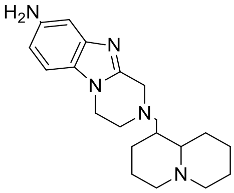

73

ID: STOCK1N-72923

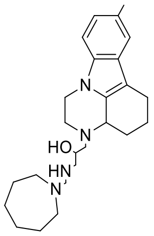

74

ID: STOCK1S-32262

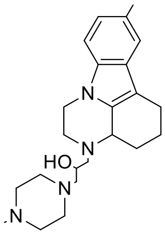

75

ID: STOCK2S-10418

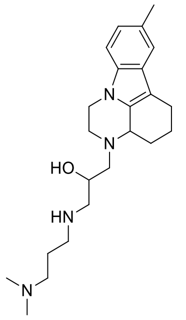

76

ID: STOCK2S-12725

References

1 Isberg, V. *et al.* Generic GPCR residue numbers - aligning topology maps while minding the gaps. *Trends Pharmacol. Sci.* **36**, 22-31, doi:10.1016/j.tips.2014.11.001 (2015).

2 Ballesteros, J. A. & Weinstein, H. Integrated methods for the construction of three-dimensional models and computational probing of structure-function relations in G protein-coupled receptors. *Methods Neurosci.* **25**, 366-428, doi:http://dx.doi.org/10.1016/S1043-9471(05)80049-7 (1995).
